# Low-Content Pre-Emulsified Safflower Seed Oil Enhances the Quality and Flavor of the *Nemipterus Virgatus* Surimi Gel

**DOI:** 10.3390/gels8020106

**Published:** 2022-02-09

**Authors:** Chunyong Song, Yufeng Lin, Pengzhi Hong, Huanming Liu, Chunxia Zhou

**Affiliations:** 1 Guangdong Provincial Engineering Technology Research Center of Marine Food, Guangdong Modern Agricultural Science and Technology Innovation Center, Guangdong Provincial Key Laboratory of Aquatic Product Processing and Safety, College of Food Science and Technology, Guangdong Ocean University, Zhanjiang 524088, China; song_li2020@163.com (C.S.); ayufenglin@163.com (Y.L.); hongpengzhi@126.com (P.H.); liu241253@gdou.edu.cn (H.L.); 2 Southern Marine Science and Engineering Guangdong Laboratory (Zhanjiang), Zhanjiang 524088, China

**Keywords:** *Nemipterus virgatus* surimi, pre-emulsified safflower seed oil, gel texture, microstructure, flavor

## Abstract

Surimi-based products occupy an important position in the aquatic product processing industry. To enhance the quality and flavor of surimi-based products, the effects of pre-emulsified safflower seed oil on the texture, water-holding capacity (WHC), microstructure, and flavor of *Nemipterus virgatus* surimi gel was evaluated. The texture and whiteness of the gel were improved, and the WHC increased (*p* < 0.05) as the content of safflower seed oil increased up to 2 mL per 100 g surimi. Furthermore, the drops of pre-emulsified safflower seed oils with an average diameter of less than 0.10 μm were evenly distributed in gel matrix. Microstructure and infrared spectroscopy analyses indicated that low-content pre-emulsified safflower seed oil acted as filler particles to occupy void spaces, resulting in gel exhibiting a dense network structure. Volatile analysis showed the gel containing pre-emulsified oil enriched volatile compounds, mainly resulting from the oxidation and decomposition of oils by the activation of lipoxygenase, which synergistically contributes to unique flavors of gel. Consequently, low-content pre-emulsified safflower seed oil can used to enhance the quality and flavor of *N. virgatus* surimi-based products. These findings are especially relevant to the current growing interest in low-fat and high-protein diets.

## 1. Introduction

Surimi, a functional ingredient used for surimi-based products, is produced by collecting fish meat and subsequent washing, dehydrating, and filtering [1,2]. Surimi-based products have been widely accepted by consumers because of their rich nutrient values, their unique gel properties, and potential as an inexpensive protein source [3,4]. During the rinsing stage of surimi production, many nutritious and valuable fish lipids are discarded to concentrate myofibrillar proteins and reduce lipid oxidation during storage. However, fish lipids play an indispensable role in maintaining the gel texture and rheological properties, providing the unique flavor of surimi-based products and generating highly nutritious surimi-based products [5]. Moreover, lipid deficiency leads to surimi gels with an extremely rough taste and unpleasant texture, severely influencing the quality and flavor of surimi-based products [6]. Therefore, the production of high-quality and unique flavors of surimi-based products has recently gained increasing attention.

Exogenous oils/fats are usually added as texture modifiers, color enhancers and processing aids during the production of surimi-based products [7]. In the current study, various animal fats and vegetable oils were added to enhance the gel texture and flavor of surimi-based products, such as camellia oil [1], fish oil [5], lard [8], coconut oil [9], and soybean oil [10]. The emulsified lard restrained the cross-linking of myosin heavy chains and destroyed the three-dimensional gel network, resulting in the poor texture and low water-holding capacity of the *Nemipterus virgatus* surimi gel [8]. Camellia oil with a high oleic acid content has been shown to effectively enhance the whiteness, texture, and sensory properties of white croaker surimi gels [1]. However, coconut oil with a high content of lauric acid significantly reduced the texture and water-holding capacity of croaker surimi gels [9]. Mi et al. [11] also found that the grass surimi gels containing 3% perilla seed oil show three-dimensional gel network structures denser than those of soybean oil and flaxseed oil. However, the water-holding capacity of gels began to decrease as the perilla seed oil content increased to 5%. Overall, the direct addition of oils can have a negative effect on surimi gels with a low water-holding capacity and high oil oxidation activity. These studies suggest that the method of adding oil should be changed to improve the gel quality.

Previous studies have confirmed that pre-emulsified oils have more positive effects on the quality of surimi gels than those containing oils without pre-emulsification [7,12]. The use of pre-emulsified virgin coconut oil with sodium caseinate resulted in oil droplets evenly distributed in the croaker surimi gels, which improved the texture and whiteness of gels, whereas this was not observed when the oil without pre-emulsification was used [13]. Silver carp surimi gels with pre-emulsified camellia seed oil had higher whiteness and water-holding capacity than gels with pre-emulsified lard, and the breaking force and deformation showed the highest values when the lard/oil content increased to 2% [14]. Similarly, the pre-emulsified lard with soybean protein isolate could also improve the whiteness and juiciness of the *N. virgatus* surimi gel, but the network structure of gels became loose and irregular as the lard content increased [8]. Consequently, pre-emulsified oil can affect the gelation properties of surimi gels, and the improvement effects may depend on the type and amount of oil. Oils with a high content of saturated fatty acids (stearic acid, palmitic acid, and lauric acid) are unfavorable to the texture properties, whereas oils with high contents of long-chain unsaturated fatty acids (oleic acid and linoleic acid) can effectively enhance the whiteness and water-holding capacity of surimi gels [11,14,15]. In addition, the high content of unsaturated fatty acids may result in the oil drop being sufficiently covered by protein and emulsified with protein to form a stable interfacial protein film, which prevents oil droplets from accumulating and decreases the range of oil distribution [16].

Safflower seed oil, known as “the king of linoleic acid”, is rich in conjugated linoleic acid, which can be fully emulsified with protein and promote the even distribution of oil droplets [16,17]. Additionally, safflower seed oil has many excellent properties such as high freezing resistance, stable fragrance, and clear color; thus, it can be used as a food processing aid [18]. Meanwhile, *N. virgatus* is an important material for surimi-based products with a high protein content and strong gel properties [8,19]. Additionally, China collected approximately 329.19 thousand tons of *N. virgatus* in 2019, showing great potential for resource development [20]. A preliminary study showed that three vegetable oils (soybean oil, corn oil, and safflower seed oil) were screened out for their beneficial gel quality and flavor, and that the low content of pre-emulsified soybean oil with milk protein could effectively improve the quality and flavor of the *N. virgatus* surimi gel [21]. Additionally, the demand for low-fat and high-protein foods is rapidly growing because an increasing number of consumers prefer low-fat healthy food [22]. Although some studies have investigated the effect of using oils on the preparation of surimi gels, there are no relevant publications about the effects of pre-emulsified safflower seed oil on the quality and flavor of surimi gels. In particular, we hypothesized that the high quality and unique flavors of surimi-based products can be produced by adding a low pre-emulsified safflower seed oil content. Therefore, the objective of this study was to explore the mechanism of improvement in the quality and flavor of *N. virgatus* surimi gel with low contents of pre-emulsified safflower seed oil. The results of this study may help elucidate the interactions between external oils and proteins of surimi gels and to develop new and functional surimi-based products.

## 2. Results and Discussion

### 2.1. Whiteness and Texture Properties Analysis of Surimi Gel

Whiteness is a significant indicator that reflects the color and quality of surimi gels and directly determines the preference of consumers [7,23]. The changes in whiteness directly reflect the changes in the three-dimensional gel network structure of surimi gels, which result from the denaturation, aggregation, and cross-linking of myofibrillar proteins [15,24]. As shown in Table 1, increasing the content of safflower seed oil resulted in an increase in the whiteness of the *N. virgatus* surimi gel (*p* < 0.05), which indicated that pre-emulsified safflower seed oil could affect interactions between protein molecules in surimi gels and change the gel network structure. When safflower seed oil was pre-emulsified with WPI, the surface of oil droplets formed an interfacial protein film for uniform distribution in the gel matrix and even suspension on the surface of surimi gels and thus led to the stronger light-scattering effect and more light reflectance in surimi gels [15,25]. Therefore, pre-emulsified safflower seed oil can significantly enhance the whiteness of *N. virgatus* surimi gel.

In the experiment, the gel strength was the maximum force value as the plunger probe was pressed down to 4 mm, and the rupture strength was the maximum force value as the plunger probe was pressed down to 10 mm. Both the gel and rupture strength can reflect the gelling ability of surimi during heating, which mainly depends on the formation and stability of the three-dimensional gel network structure [10]. Compared with the control, the gel strength, rupture strength, hardness, adhesiveness, cohesiveness, and chewiness of surimi gels with pre-emulsified safflower seed oil were improved until the safflower seed oil content increased up to 2 mL per 100 g of surimi (*p* < 0.05). This demonstrated that the low content of pre-emulsified safflower seed oil had positive effects on the gel texture, while high contents were adverse for gel texture. These results agree with those reported by Kang, Chen, and Ma [26], who found that low-fat frankfurter sausage with pre-emulsified soybean oils had stronger texture properties. It is known that there is a linear relationship between the protein content and texture of surimi gels [10]. Myofibrillar proteins directly participate in the formation of the three-dimensional gel network structure of surimi gels. At the same moisture level, the increase in pre-emulsified safflower seed oil content leads to a corresponding decrease in myofibrillar protein content in surimi gels, which results in a loose network structure with weak gel strength and rupture strength [10]. However, WPI has a stronger emulsifying ability and penetrating ability than myofibrillar proteins. Thus, during the gelation process of surimi paste, myofibrillar protein is less likely to participate in emulsification with safflower seed oil and tends to form a three-dimensional gel network structure, which enhances the texture of surimi gels. Whey protein concentration contains a cathepsin inhibitor to inhibit the deterioration of myosin and has been shown to enhance the texture of goatfish surimi gels [27]. Therefore, appropriate contents of pre-emulsified safflower seed oil can effectively modify the texture of surimi gels. In this study, the gel containing 2 mL of safflower seed oil per 100 g of surimi had the strongest texture properties.

### 2.2. WHC and CLR Analysis of Surimi Gel

**Figure 1:**
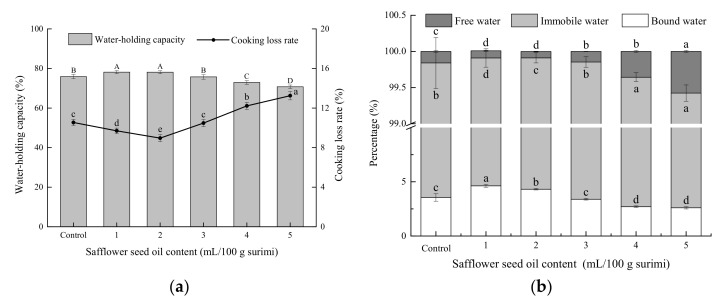
Effect of pre-emulsified safflower seed oil on the WHC, CLR (**a**), and moisture content (**b**) of the *N. virgatus* surimi gel.

During the gelation process of surimi paste, myofibrillar proteins are heat-induced and form a three-dimensional gel network structure to trap free water in the gel matrix and to make surimi gels possessing a high WHC [15,19,24]. Similarly, the CLR is the percentage of the quality of water, oil, and other substances that easily leak out during the cooking process, accounting for the quality of surimi gels [28]. The WHC and CLR are important quality parameters for evaluating the stability of surimi gels, which reflects the ability of the three-dimensional gel network structure to retain water. A high WHC and low CLR indicate that the gels can trap large quantities of water. The addition of safflower seed oil resulted in a decrease in the WHC and an increase in the CLR of surimi gels (*p* < 0.05) (Figure S1). However, the WHC of surimi gels with pre-emulsified safflower seed oil initially increased and then decreased with increasing oil content. The WHC values were the highest at 2 mL of safflower oil per 100 g of surimi (Figure 1a). Similarly, the CLR initially decreased and then increased as the content of safflower seed oil increased. In this case, the differences in the WHC and CLR of gels with the pre-emulsified safflower seed oil suggested that pre-emulsification could change the interaction between the oil droplets and proteins, thus affecting the formation of the three-dimensional gel network structure. Similar results were obtained by Zhou et al. [14], who found that silver carp surimi gels with pre-emulsified lard/camellia seed oils had a higher WHC than those with the oils without pre-emulsification. Song et al. [21] also found that *N. virgatus* surimi gel with pre-emulsified soybean oil could trap more water and thus had a higher WHC.

Pre-emulsification can form an interfacial protein film on the surface of the oil droplets. Different contents of pre-emulsified safflower seed oil produce different emulsifying effects, resulting in a different WHC and CLR of gels. These differences may be related to the filling properties, size, and distribution of oil droplets. The low contents of pre-emulsified safflower seed oil show a stronger interfacial protein film on the oil droplet surface to hydrate it with water molecules and fill in the gel matrix. This effect results in more water being wrapped in the gel matrix, which enhances its physical stability and binding ability. In addition, low contents of pre-emulsified oil can also form smaller oil droplets and distribute them more evenly in the gel matrix [7]. These combined effects led to an increase in the WHC and a decrease in the CLR of surimi gels (*p* < 0.05). In contrast, the high content of pre-emulsified oil can interfere with the interaction between protein molecules by increasing the distances between protein molecules, leading to gels with a loose and uneven three-dimensional gel network structure [7,29]. In addition, excessive safflower seed oil may occupy the void spaces of water molecules in the gel matrix and prevent water migration from the gel network, leading to a decrease in the WHC of surimi gels [1]. These results indicate that the low-content pre-emulsified safflower seed oil is beneficial for the formation of the three-dimensional network structure of surimi gels. The optimal safflower seed oil content determined by this study was 2 mL per 100 g of surimi.

### 2.3. Moisture Distribution and Composition

LF-NMR is the most direct and effective method to determine the moisture distribution and composition of surimi gels [28]. The moisture state in gels directly affects the WHC and further determines the quality and stability of surimi gels. Immobilized water in surimi gels is the main form of water and depends on the change in the gel network structure [28]. In addition, immobilized water is an important indicator that reflects the WHC of surimi gels. As shown in Figure 1b, with the increase in safflower seed oil content, the free-water content increased (*p* < 0.05), and the bound-water content decreased (*p* < 0.05). However, compared with the control, the gels containing the lowest contents of pre-emulsified safflower seed oil exhibited lower contents of free water (*p* < 0.05) and higher contents of bound water (*p* < 0.05). The low content of pre-emulsified oil reduced the damage to the network structure due to small oil droplets and uniform distributions in the gel matrix [7]. Additionally, the polar groups on the surface of WPI contain a hydrophilic effect, permitting free water and immobilized water to tightly combine with the protein and then transform into bound water, which increases the WHC of surimi gels. However, a high oil content increases the distances between protein molecules, which damages the density and uniformity of the network structure and occupies the void spaces of water molecules [1]. This increases the mobility of immobilized and bound water, permitting them to detach from the tissue structure and transform into free water, which decreased the WHC of surimi gels. Thus, safflower seed oil influenced the moisture distribution during the gelatinization of surimi paste, while a low content of pre-emulsified safflower seed oil can reinforce immobilized water in surimi gels. The results confirm that a low content of pre-emulsified safflower seed oil is beneficial for retaining water in surimi gels. It has also been reported that a large number of water molecules are restricted in the gel network; thus, the WHC of gels increases with an increase in the immobilized water content [30,31,32].

### 2.4. Oil Droplet Diameter Size Distribution

**Figure 2:**
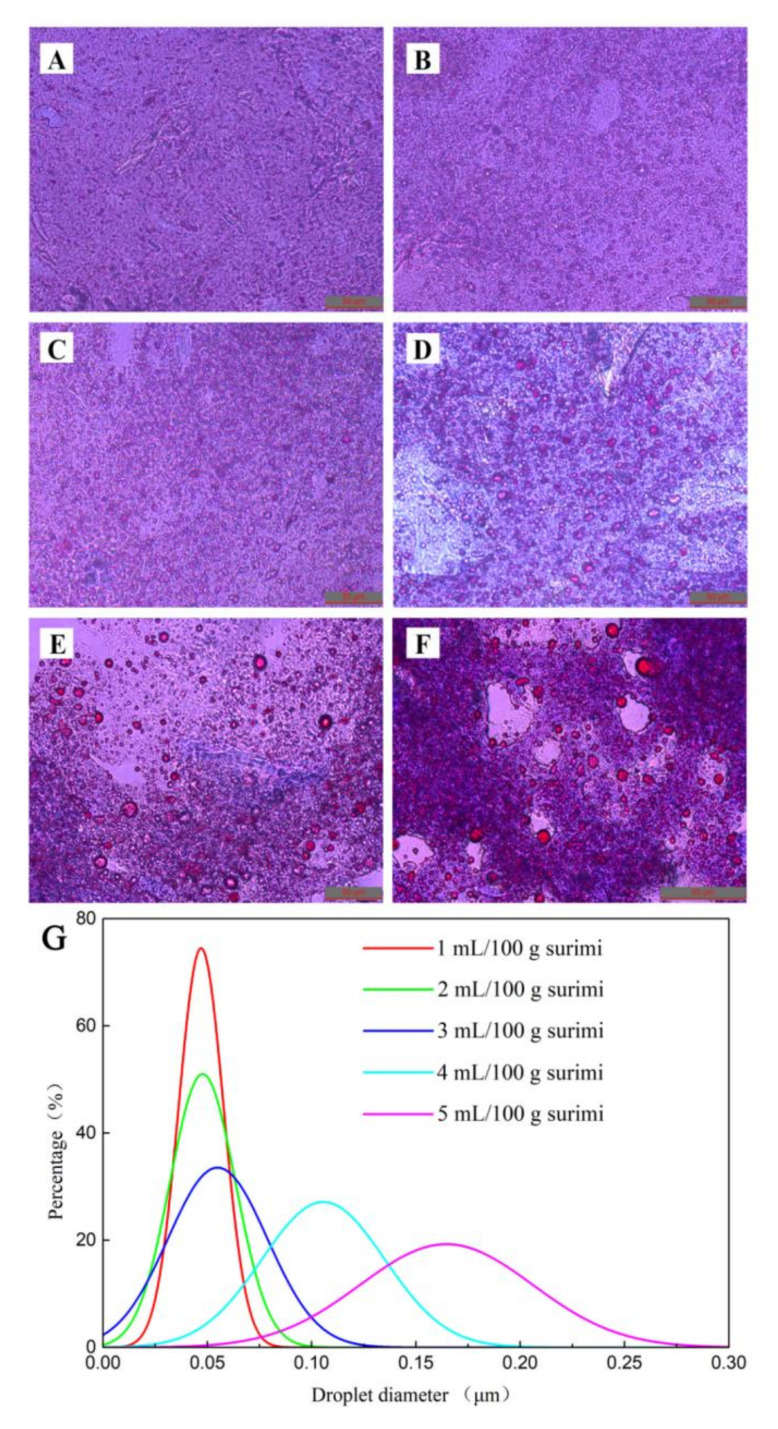
Effects of pre-emulsified safflower seed oil on the oil droplet distribution (×400) of the *N. virgatus* surimi gel. (**A**): control; (**B**–**F**): the surimi gels containing 1, 2, 3, 4, and 5 mL of safflower seed oil per 100 g surimi, respectively; (**G**): pre-emulsified safflower seed oil droplet diameter distribution image.

To identify the distribution and aggregation of oil droplets in surimi gels, optical microscope image analysis was performed. The surface of the *N. virgatus* surimi gel was relatively smooth, and oil droplets were rarely observed (Figure 2A). After adding pre-emulsified safflower seed oil, obvious traces of oil droplets were observed in the surimi gels. When the content of safflower seed oil was not higher than 2 mL per 100 g of surimi, the small oil droplets were evenly distributed in the network structure and embedded in the gel matrix (Figure 2B,C). Furthermore, the diameter of oil droplets was mainly in the range of 0.25–0.75 μm (Figure 2G). This result further showed that the low-content pre-emulsified safflower seed oil with WPI could be evenly distributed in the network structure of surimi gels in the form of small particles. Similarly, pre-emulsified peanut oil is evenly distributed in the gel matrix [7]. However, with the increase in safflower seed oil content, the distribution of oil droplets became increasingly uneven, and the average diameter of the oil droplets increased. When the content of safflower seed oil increased to 5 mL per 100 g of surimi, oil droplets with a diameter greater than 0.10 μm accumulated in the gel matrix (Figure 2F). When the number of oil droplets is excessive, they merge into larger oil droplets to decrease the interface energy, which eventually interferes with the formation of a gel network structure during heating [7,33]. Additionally, the large, formed oil droplets further promote the accumulation of oil droplets. Gani and Benjakul [13] also proved that large oil droplets could interfere with protein interactions by increasing the distance between protein molecules. Based on these reports, we hypothesized that the low content of pre-emulsified safflower seed oil was evenly distributed in the gel matrix, which reduced the interference of oils on myofibrillar protein and further enhanced the physical stability of surimi gels.

### 2.5. Microstructure of Surimi Gel

**Figure 3:**
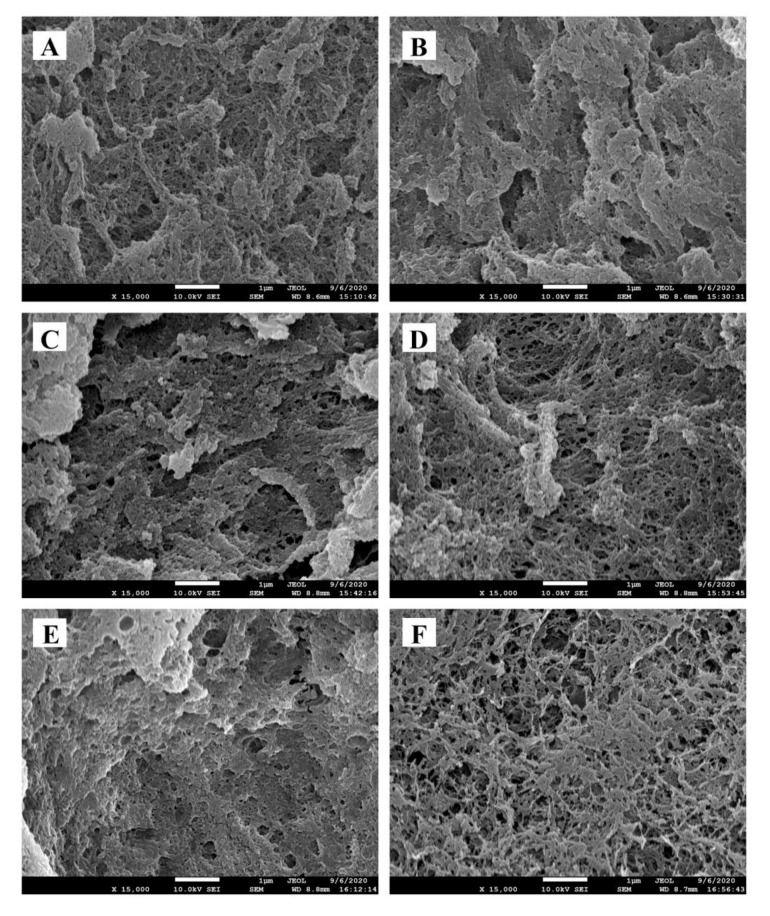
Effects of pre-emulsified safflower seed oil on the SEM (×15,000) of the *N. virgatus* surimi gel. (**A**): control; (**B**–**F**): the surimi gels containing 1, 2, 3, 4, and 5 mL of safflower seed oil per 100 g surimi, respectively.

The microstructure of the *N. virgatus* surimi gel was determined using SEM (Figure 3). Compared with the control (Figure 3A), the surimi gels with low contents of pre-emulsified safflower seed oil show a network structure with fewer holes and a smaller size (Figure 3B,C). This may be associated with the uniform distribution and reduced interference of small oil droplets in the gel matrix. Moreover, the pre-emulsified safflower seed oil may also play a role, as filler particles occupy the void spaces in the three-dimensional gel network structure of surimi gels [9]. This result is consistent with the stronger texture, higher WHC, and lower CLR of surimi gels containing low-content pre-emulsified safflower seed oil (Table 1 and Figure 1A). However, as the content of safflower seed oil increased from 3 to 5 mL per 100 g of surimi, the microstructure became loose with large holes (Figure 3D–F). These results are similar to the microstructure of surimi gels with camellia tea oil, as observed by Zhou et al. [1]. Excessive oil droplets accumulate together to form large oil droplets, which negatively impacts protein intermolecular interactions by increasing the distance between protein molecules [1,13]. Surimi gels containing high contents of pre-emulsified safflower seed oil exhibited a loose network structure with a weak texture and low WHC. The results further prove that the low-content pre-emulsified safflower seed oil was conducive to the formation of the dense gel network structure.

### 2.6. FT-IR Spectral Analysis of Safflower Seed Oil and Surimi Gel

**Figure 4:**
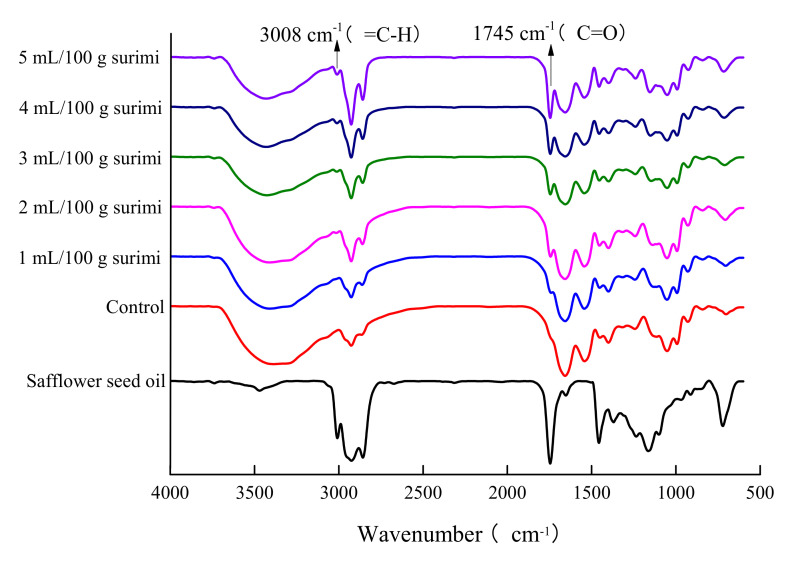
FT-IR spectra of safflower seed oil and the *N. virgatus* surimi gel.

FT-IR spectroscopy is an effective method of determining the conformational changes of proteins. The FT-IR spectra of safflower seed oil and *N. virgatus* surimi gel within the range of 4000–600 cm^−1^ are shown in Figure 4. The control and surimi gels with different contents of pre-emulsified safflower seed oil had similar characteristic adsorption bands. However, compared with the control, the surimi gels with pre-emulsified safflower seed oil presented distinct strong adsorption peaks at 3008 cm^−1^ and 1745 cm^−1^, attributed to the changes in the stretching vibration of =C–H and stretching vibration of C=O in safflower seed oil [24,34]. The changes in these new absorption peaks are consistent with the FT-IR spectra of safflower seed oil. The amide I band is a widely used region for analyzing the changes in the secondary structure of proteins. This band includes the wavelength ranges of 1650–1660, 1665–1680, 1660–1695, and 1660–1665 cm^−1^, which originate from the structures of α-helices, β-sheets, β-turns, and random coils, respectively [24]. However, with the increase in safflower seed oil content, there were no changes in the amide A, amide I, amide II, amide III, or other feature absorption peaks of protein structure in surimi gels, which further indicated that there was no significant interaction between the pre-emulsified safflower seed oil and the myofibrillar proteins during the gelatinization process of surimi. The results are consistent with previous research reporting that the oils had no effect on protein but acted as filler particles to occupy the void spaces in gels [1,14]. However, some studies have reported that oil can change the conformation and local chemical environment of proteins by inducing the exposure of hydrophobic groups in protein molecules [5]. For example, the accumulation of fish oil has been reported to hinder the rearrangement and unfolding of proteins and affect the hydration of proteins [24,33]. This difference may be related to the type and amount of oil added. In this study, the pre-emulsified safflower seed oil acted as filler particles to occupy the void spaces of surimi gels.

### 2.7. Lipid Oxidation and Lipoxygenase Activity of Surimi Gel

**Figure 5:**
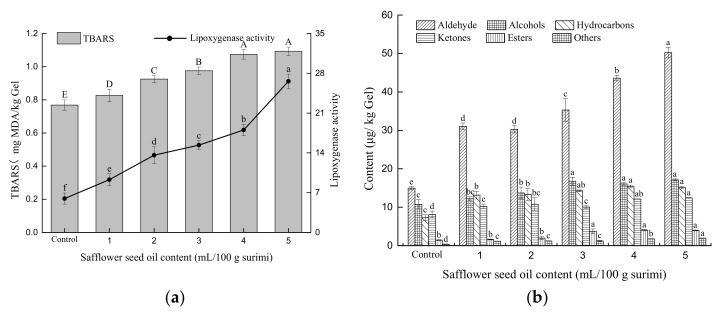
Effect of pre-emulsified safflower seed oil on the TBARS content, lipoxygenase activity (**a**), composition, and content in volatile compounds (**b**) of the *N. virgatus* surimi gel.

Malondialdehyde (MDA) is the main secondary oxidation product of oil and can be used to evaluate the degree of oil oxidation and rancidity [25]. The changes in MDA content are positively correlated with the degree of oil oxidation. The MDA content increases with the increase in oil oxidizability, which leads to an increase in the TBARS content. Although pre-emulsification can form an interfacial protein film on the surface of oil droplets, safflower seed oil is rich in unsaturated fatty acids, and its linoleic acid content is greater than 67.8%. Thus, it is easily oxidized to produce MDA during heating. As shown in Figure 5a, the TBARS content of the *N. virgatus* surimi gel increased (*p* < 0.05) with an increase in the safflower seed oil content. Aheto et al. [35] reported that the maximum TBARS content was 4.75 mg MDA/kg in dried pork products. Berruga, Vergar, and Gallego [36] suggested that an acceptable level of TBARS contents is 4.2–7.5 mg MDA/kg in meat products. In this experiment, the TBARS content in the *N. virgatus* surimi gel containing pre-emulsified safflower seed oil was less than 1.2 mg/kg gel (Figure 5a), which is far below the acceptable range. Therefore, pre-emulsified safflower seed oil can be used as a processing aid to enhance the gel quality of surimi gels, but necessary antioxidant measures should be considered to improve the storage stability of surimi gels.

Lipoxygenase is a class of non-heme iron-containing enzymes that can specifically catalyze polyunsaturated fatty acids to produce a series of fatty acid hydroperoxides [37]. These hydroperoxides further decompose to form small-molecular compounds, such as alcohols, aldehydes, ketones, and their alcoholic counterparts [38]. Some small-molecule compounds contribute to volatile flavor components in food systems. In addition, the activity of lipoxygenase is positively correlated with a better flavor of the surimi gels. The effect of pre-emulsified safflower seed oil on the lipoxygenase activity of *N. virgatus* surimi gel is shown in Figure 5a. With the increase in safflower seed oil content, the activity of lipoxygenase becomes stronger, which indicates that pre-emulsified safflower seed oil could change the flavor of surimi gels. The safflower seed oil in the gel matrix may activate certain enzymes, such as lipoxygenase, lipase, and peroxidase, which attack unsaturated fatty acids and produce hydroperoxides and volatiles.

### 2.8. Volatile Compounds of Surimi Gel

The volatile components of the gels were analyzed using by HS-SPMEGC-MS. The *N. virgatus* surimi gel contained 48 volatile compounds. The surimi gels with 1, 2, 3, 4, and 5 mL of safflower seed oil per 100 g of surimi contained 56, 57, 62, 60, and 61 types of volatile compounds, respectively (Table S1). These changes indicated that the pre-emulsified safflower seed oil significantly increased the number of volatile components in surimi gels. Safflower seed oil is rich in unsaturated linoleic acid, which is easily oxidized and decomposed to form small-molecular compounds during heating. Thus, aldehydes, alcohols, ketones, hydrocarbons, and esters were dominant in the volatile compounds of gels, which synergistically gave a unique flavor to surimi-based products.

As shown in Figure 5b, the most abundant volatile compounds in the *N. virgatus* surimi gel were aldehydes. The aldehyde content was 14.94 μg/kg, which represented 34.95% of the total volatiles in the control. After adding pre-emulsified safflower seed oil, the changes in aldehydes were the most obvious (*p* < 0.05), and its content increased as the content of safflower seed oil increased. Among the aldehydes, hexanal, heptanal, benzaldehyde, octanal, nonanal, and decanal were detected at high concentrations. These aldehydes have a low threshold with a strong pungent odor. However, as the number of carbon atoms increases, the pungent odor becomes weak and gradually presents a pleasant odor, originating from a typical unsaturated fatty acid oxidation product that is a key component of the fish flavor [39].

Alcohols, which can present brisk and soft odors, are an important part of the flavor in the *N. virgatus* surimi gel. Eight types of alcohol compounds, including 1-octen-3-ol, 2-ethyl-1-hexanol, 3-cyclohexene-1-ethanol, and phenethyl alcohol, were detected. The abundancy of these alcohols was 10.76 μg/kg, which represented 25.00% of the total volatiles in the control. 1-octen-3-ol was detected at the highest concentrations among the alcohols (*p* < 0.05), and its threshold was low with the smell of mushroom and soil mold. Thus, it is one of the volatile compounds with the highest flavor activity in *N. virgatus* surimi gel. In addition, it is mainly produced by the action of lipoxygenase on linoleic acid hydroperoxide decomposition [40]. The content of 1-octen-3-ol increased as the content of safflower seed oil increased.

The most common compounds in the *N. virgatus* surimi gel were hydrocarbons formed by the oxidation of fatty acids. A total of 23 distinct types of hydrocarbons were detected. Although the hydrocarbon compounds detected were tasteless with high threshold [41], hydrocarbons, especially unsaturated hydrocarbons, can be oxidized to produce flavor compounds such as alcohols and aldehydes ketones, among others. Therefore, hydrocarbons also have an indispensable contribution to the flavor of surimi gels.

Ketones are carbon-based compounds with a low flavor threshold value that can produce a native and rich fragrance smell and thus have a significant contribution to the flavor of gels [42]. In this study, 2,5-octanedione was the main ketone detected in the control (5.16 μg/kg). This compound had a fruity and milky aroma. The content of 2,5-octanedione increased as the content of safflower seed oil increased (*p* < 0.05).

Only a few types of esters were found in low abundancy in the *N. virgatus* surimi gel. Most of the detected esters were high-molecular weight esters. As the molecular weight of esters increases, the fruity fragrance smell is lower, and the threshold is higher [42]. Therefore, esters contributed little to the flavor of the *N. virgatus* gel. Other types of compounds detected were mainly nitrogen-containing compounds, which are small-molecule compounds resulting from the decomposition of amino acids and proteins. Since only a few types at low concentrations were found, we hypothesize that they barely contributed to the flavor of the gels.

The overall analysis indicated that aldehydes, alcohols, and ketones were the main components of flavor, and that hydrocarbons were important precursor compounds for the formation of these flavor substances. Therefore, after adding pre-emulsified safflower seed oil, the types and contents of volatile components, including aldehydes, alcohols, hydrocarbons, and ketones, increased in surimi gels (*p* < 0.05), which synergistically contributed to the unique flavor of the *N. virgatus* surimi gel, especially when the safflower seed oil content reached 2 mL per 100 g of surimi or more.

## 3. Conclusions

The addition of pre-emulsified safflower seed oil had an obvious effect on the quality and flavor of the *N. virgatus* surimi gel. The low contents of pre-emulsified safflower seed oil were evenly distributed in the gel matrix as small oil droplets, and the gels exhibited a dense three-dimensional gel network structure with a high WHC. Moreover, pre-emulsified safflower seed oil in the gel matrix could activate lipoxygenase, resulting in gels with pre-emulsified oils producing a large number of volatile components. Among them, aldehydes and hydrocarbons were the most common compounds, and alcohols and ketones were an important part of the flavor. The results reveal insight into the enhancement mechanism of surimi gels with low contents of pre-emulsified safflower seed oil. There was no significant interaction between the pre-emulsified safflower seed oil and the myofibrillar proteins, but the low-content pre-emulsified safflower seed oil acted as filler particles to occupy the void spaces and gave a unique flavor to gels. The present results confirm our hypothesis that the high quality and unique flavors of surimi-based products can be produced by adding low pre-emulsified safflower seed oil content. These findings are especially relevant to the current growing interest in the development of low-fat and high-protein diets.

## 4. Materials and Methods

### 4.1. Materials and Reagents

Frozen *N. virgatus* surimi (AAA-grade, moisture content: 73.96 ± 0.26%) was purchased from Fenghua Food Co., Ltd. (Beihai, China) and stored at −20 °C. Safflower seed oil containing more than 67.8% linoleic acid was purchased from COFCO Tayuan Honghua Co., Ltd. (Xinjiang, China). Whey protein isolate (WPI, food grade) was purchased from Weifeng Biological Technology Co., Ltd. (Zhengzhou, China). Tissue-Tek O.C.T. was purchased from Sakura Finetek Japan Co., Ltd. (Tokyo, Japan). Tween 20 was purchased from Aladdin Biochemical Technology Co., Ltd. (Shanghai, China). Linoleic acid (≥95%, GC) and 2-methyl-3-heptanone (≥95%, GC) were purchased from Macklin Biochemical Co., Ltd. (Shanghai, China). The N-alkane mix was purchased from Tan-Mo Technology Co., Ltd. (Changzhou, China). Ethylenediaminetetraacetic acid (EDTA) was purchased from the Chemical Factory Co., Ltd. (Changzhou, China). The remaining chemical reagents used in the experiments were of analytical grade and purchased from the Chemical Reagent Factory (Guangzhou, China).

### 4.2. Preparation of Pre-Emulsified Safflower Seed Oil

Pre-emulsified safflower seed oil was prepared according to a previously described method [13] with some modifications. WPI solution (2% protein, *w*/*v*) was prepared by mixing WPI (1.6 g) with deionized water (80 mL). Then, 16 mL of the WPI solution was mixed with safflower seed oil in different volumes (3, 6, 9, 12, and 15 mL). The mixtures were intermittently homogenized at 11,000 rpm for 5 min during 5 cycles using a homogenizer (T8DS25; IKA Laboratory Equipment, Staufen, Germany) to obtain different pre-emulsified safflower seed oils. Each cycle was 1 min for homogenization and 5 min for intermission.

### 4.3. Preparation of Surimi Gel

After thawing at 4 °C overnight, surimi was cut into small pieces. Salts (2.5 g/100 g) were added to surimi and chopped at a speed of 2100 rpm for 2 min in a Stephan vertical vacuum cutter (Model UM 5; Stephan Machinery Co., Hameln, Germany). Subsequently, different pre-emulsified safflower seed oils were added to salted surimi pastes, and the final moisture content was adjusted to 80% with ice water and chopped at the speed of 2100 rpm for 3 min. The final content of safflower seed oil in surimi was 1, 2, 3, 4, and 5 mL per 100 g of surimi, respectively. During chopping, water was used as the cooling medium to maintain the sample temperature below 8 °C. After eliminating the air pockets, surimi was poured into a plastic casing (diameter, 2.5 cm), and both ends were sealed. Finally, the samples were incubated at 40 °C for 30 min and then in a water bath at 90 °C for 20 min [1,13]. Then, samples were immediately placed in ice water and stored at 4 °C. Surimi gel without pre-emulsified safflower seed oil was used as the control.

### 4.4. Whiteness Evaluation

After equilibrating at room temperature (25 °C) for 1 h, the whiteness of the samples was evaluated using a colorimeter (Model NS800; 3NH Technology Co., Ltd., Shenzhen, China). The parameters L* (lightness), a* (red/green), and b* (yellow/blue) were measured in five replicates, and the average values were calculated. The whiteness was calculated using the following Equation (1) [14]:

(1) $$Whiteness\;=100-\sqrt{{\left(100-L*\right)}^{2}+{a*}^{2}+{b*}^{2}}$$

### 4.5. Texture Properties of Gel

Texture properties analysis (TPA) of gels were performed following the method of [5] with some modifications. TPA measurement mode and gel strength measurement mode of texture analyzer (Model TA.XT plusC; STab. Micro System Ltd., Surrey, UK) were used with P/0.5S spherical plunger probes and P/0.5 flat plunger probes, respectively. After equilibrating at room temperature (25 °C) for 30 min, the plunger probe was pressed perpendicularly into the cross-section of the sample at a speed of 1.00 mm/s. Other test parameters were as follows: pre-test and return speed, 1.00 mm/s; trigger force, 5 g; and compression strain, 50%. Subsequently, textural parameters (hardness, adhesiveness, springiness, cohesiveness, gumminess, chewiness, and resilience) were calculated using Texture Expert version 1.22. Each sample was measured in five replicates, and the average values were calculated.

### 4.6. Water-Holding Capacity (WHC)

The WHC was measured following the method of [1]. Samples were chopped into bite-sized pieces (5 mm × 5 mm × 5 mm) and weighed accurately (*M*_1_). Samples were wrapped with two filter papers and centrifuged (J-26sxp; Avanti, Beckman, Indianapolis, IN, USA) at 10,000 rpm for 10 min. After centrifugation, the samples were weighed again (*M*_2_). The WHC was calculated using the following Equation (2) and expressed as a percentage of the sample weight:

(2) $$\mathrm{WHC}/\%=\frac{M_{2}}{M_{1}}\times 100$$

### 4.7. Cooking Loss Rate (CLR)

The CLR was measured according to the method of [26]. Samples were cut into small cylinders and weighed accurately (*G*_1_). Subsequently, the samples were sealed with a cooking bag and placed in a water bath at 90 °C for 20 min. After cooking, the liquid on the gel surface was dried with filter paper, and the samples were weighed again (*G*_2_). The CLR was calculated using the following Equation (3) and expressed as a percentage of the sample weight:

(3) $$\mathrm{CLR}/\%=\frac{G_{1}-G_{2}}{G_{1}}\times 100$$

### 4.8. Moisture Distribution and Composition

Moisture distribution and composition in surimi gels were determined according to the method of [5] with some modifications. Briefly, surimi gels were cut into small cylinders (40 mm × 15 mm × 15 mm) and placed in an NMR tube with a diameter of 40 mm. The transverse relaxation time (*T*_2_) was determined using the Carr–Purcell–Meiboom–Gill (CPMG) sequence of the NMR analyzer (NMI20-060H-I; Niumag Co., Ltd., Suzhou, China). Each peak area in integral spectrum of *T*_2_ was accumulated for calculation of water distribution and composition in surimi gels.

### 4.9. Light Microscopic Images Analysis

The distribution of oil droplets was analyzed according to the method of [7] with some modifications. After dehydration with 30% sucrose, surimi gels were embedded and fixed with Tissue-Tek O.C.T. The samples were then cut into 20 μm-thick slides using a microtome (Leica CM1950; Leica Microsystems Ins., Nussloch, Germany). Subsequently, the samples were dyed with 1% bromophenol blue solution (protein dye) and 0.1% Sudan IV dye solution (oil dye) for 1 min, and the excess dye was washed with distilled water. The distribution of oil droplets in surimi gels was observed using an Olympus microscope (CKX41; Olympus Optical Co., Ltd., Tokyo, Japan) at a magnification of 400×. ImageJ software was used to measure the droplet diameter and then draw the droplet diameter distribution image.

### 4.10. Scanning Electron Microscopy (SEM)

The microstructure of the gel samples was analyzed using an SEM (7610F; Japan Electronics Co., Ltd., Tokyo, Japan). Samples were cut into 1 mm-thick pieces, fixed with glutaraldehyde solution, washed with phosphate buffer, successively dehydrated with ethanol, replaced by tert-butanol, freeze-dried, and sputter-coated with gold [24]. The microstructure of the samples was observed at an acceleration voltage of 8 kV and a magnification of 15,000×.

### 4.11. Fourier-Transform Infrared Spectroscopy (FT-IR) of Surimi Gel

Surimi gels were freeze-dried and comminuted. The powder and potassium bromide (KBr) were mixed at a ratio of 1:100 and then compressed into a 1 mm pellet. For safflower seed oil, after compressing into a 1 mm pellet with KBr, a small drop of safflower seed oil was dropped on it for measurement. The transparent pellet was scanned by infrared spectra (TENSOR27; Bruker Ltd., Ettlingen, Germany) with a scanning wavelength range of 4000–600 cm^−1^, and samples were scanned 16 times at a resolution of 4 cm^−1^ [23].

### 4.12. Lipid Oxidation of Surimi Gel

Thiobarbituric acid reaction substances (TBARS) were used to evaluate the degree of lipid oxidation in surimi gels. Malondialdehyde (MDA) content in the gels was determined according to the method of [25,35] with some modifications. Surimi gels (5.0 g) were mixed with 7.5% trichloroacetic acid (50 mL) containing 0.1% EDTA and heated at 50 °C for 30 min. The mixtures were filtered using two layers of filter paper. The filtrate (2 mL) was mixed with 0.02 M thiobarbituric acid solution (2 mL) and heated at 90 °C for 30 min. After cooling, the absorbance was measured at 532 nm using a UV-Vis spectrophotometer (Cintra 1010; GBC Scientific Equipment Pty Ltd., Sydney, Australia). The MDA standard curve was formulated from 1,1,3,3-tetraethoxypropane, and TBARS content was expressed as the mass of the MDA equivalent per kilogram of gel (mg/kg).

### 4.13. Lipoxygenase Activity of Surimi Gel

Lipoxygenase activity was determined according to the method of [37] with some modifications. Briefly, 50 mM phosphate-buffered solution (pH 7.4, 1.0 mM dithiothreitol, and 1.0 mM EDTA) was mixed with gel samples at a ratio of 4:1 (*v/w*) and then homogenized at 15,000 rpm in an ice bath for 1 min. After filtering through four layers of gauze, the mixtures were centrifuged at 10,000 g for 30 min. The supernatant was regarded as a lipoxygenase crude enzyme solution for further analysis. For the substrate solution, linoleic acid (140 µL) was mixed with Tween 20 (180 µL) and emulsified in 10 mL of deoxygenated redistilled water. The pH of the mixtures was adjusted to 9.0, with 2 M NaOH, and diluted with deoxygenated redistilled water to 50 mL. The activity of lipoxygenase was determined by measuring the increase in the absorbance of the substrate solution at a wavelength of 234 nm using a UV-Vis spectrophotometer (Cintra 1010; GBC Scientific Equipment Pty Ltd., Sydney, Australia) at room temperature (25 °C). A total of 0.1 mL of enzyme solution was added to a quartz cell containing 50 mM citric acid solution (2.9 mL, pH 5.5) and 0.2 mL linoleic acid substrate solution quickly, and the increase in absorbance was recorded within 1 min. The unit of lipoxygenase activity was U/g, where U was defined as an increase in absorbance of 0.001 per minute. The control consisted of 0.2 mL of linoleic acid substrate solution and 3.0 mL of citric acid buffer solution. Each sample was measured five times, and the average values were calculated.

### 4.14. Volatile Compounds of Surimi Gel

Volatile compounds in surimi gels were detected using an SH-Rxi-5Sil MS capillary column according to the method of headspace solid-phase microextraction–gas chromatography-mass spectrometry (HS-SPME-GC-MS) [41,43]. Ten grams of each sample was chopped and poured into a 20 mL headspace vial. After the samples were incubated at 60 °C for 10 min, the SPME (50/30 μm DVB/Carbowxen on PDMS) was exposed for extracting volatile compounds at 60 °C for 30 min. Subsequently, the fiber was inserted into the GC injector port quickly and heated at 250 °C for 3 min. The heating procedure was set as follows: the original temperature was set at 50 °C and kept for 5 min, then increased to 100 °C at a speed of 5 °C/min and maintained for 2 min. Subsequently, the temperature was increased to 140 °C at a speed of 4 °C/min and maintained for 1 min and then increased to 180 °C at the same speed. The temperature was maintained at 180 °C for 2 min, and then the temperature was increased to 250 °C at a speed of 5 °C/min and maintained for 5 min. Helium was supplied as a carrier gas at a flow rate of 1.5 mL/min in splitless mode. The mass spectrometer parameters were set as follows: ion source temperature, 230 °C; ionization energy, 70 eV; acquisition mode, full-scanning mode; and data collection over the *m*/*z* range of 35–550 amu. Qualitative analysis was performed using a computer to search for each compound and match it with the NIST 14 library database. When the matching degree was more than 80 (maximum value 100), the compounds were regarded as identified. Simultaneously, under the same chromatographic conditions, the n-alkane mix was used to analyze the retention index (RI) of the matched volatile compounds. The specific calculation formula is as follows is given as Equation (4). In the equation, n is the number of carbon atoms of the tested volatile compound, *t*_χ_ is the retention time of the tested volatile compound, *t*_n_ is the retention time of n-alkane with the same number of carbon atoms as the tested volatile compound, and *t*_n+1_ is the retention time of n-alkane with one more carbon atom than the volatile compounds. The absolute calibration factor of volatile compounds was set to 1.0.

Based on the results of MS and RI identification and using 2-methyl-3-heptanone as an internal standard and its mass concentration, the peak area of each volatile compound was compared with the peak area of the internal standard to analyze volatile compounds in surimi gels. (The absolute calibration factor of volatile compounds was set to be 1.0).

(4) $$RI=100\times (\frac{t_{\chi }}{t_{n+1}-t_{n}})+100n$$

### 4.15. Statistical Analysis

All experiments were independently performed in triplicate with numerous different samples. Statistical analysis (ANOVA and Duncan’s multiple range test) was performed using SPSS software (version 19.0; SPSS Inc., Chicago, IL, USA). All figures are expressed as the mean ± standard deviation (SD).

## Figures and Tables

**Table 1:** Effect of pre-emulsified safflower seed oil on whiteness and texture properties of the *Nemipterus virgatus* surimi gel.

Properties	Control	Safflower Seed Oil Content (mL/100 g Surimi)				
		1	2	3	4	5
Whiteness	68.22 ± 0.10 ^f^	70.30 ± 0.27 ^e^	70.81 ± 0.04 ^d^	71.62 ± 0.04 ^c^	73.77 ± 0.43 ^b^	74.39 ± 0.13 ^a^
Gel strength (N)	4.890 ± 0.078 ^b^	5.067 ± 0.055 ^a^	5.134 ± 0.087 ^a^	4.906 ± 0.084 ^b^	4.645 ± 0.084 ^c^	4.301 ± 0.087 ^d^
Rupture strength (N)	13.280 ± 0.098 ^c^	13.812 ± 0.056 ^b^	14.178 ± 0.134 ^a^	13.173 ± 0.116 ^c^	12.475 ± 0.281 ^d^	11.476 ± 0.276 ^e^
Hardness (N)	9.982 ± 0.123 ^b^	10.065 ± 0.116 ^b^	10.645 ± 0.228 ^a^	9.589 ± 0.107 ^c^	9.272 ± 0.054 ^d^	8.952 ± 0.142 ^e^
Adhesiveness (g·s)	2.058 ± 0.040 ^c^	2.430 ± 0.070 ^b^	3.093 ± 0.103 ^a^	2.417 ± 0.069 ^b^	2.047 ± 0.048 ^c^	1.833 ± 0.056 ^d^
Springiness	0.606 ± 0.004 ^b^	0.593 ± 0.004 ^c^	0.581 ± 0.005 ^d^	0.611 ± 0.004 ^b^	0.622 ± 0.004 ^a^	0.624 ± 0.005 ^a^
Cohesiveness	0.589 ± 0.006 ^ab^	0.583 ± 0.009 ^b^	0.591 ± 0.007 ^a^	0.555 ± 0.005 ^c^	0.538 ± 0.003 ^d^	0.520 ± 0.004 ^e^
Gumminess	5.919 ± 0.082 ^a^	5.956 ± 0.157 ^a^	6.041 ± 0.181 ^a^	5.556 ± 0.062 ^b^	5.286 ± 0.090 ^c^	5.021 ± 0.076 ^d^
Chewiness	3.792 ± 0.047 ^b^	4.135 ± 0.140 ^a^	4.093 ± 0.086 ^a^	3.636 ± 0.048 ^c^	3.396 ± 0.037 ^d^	3.236 ± 0.093 ^e^
Resilience	0.221 ± 0.005 ^d^	0.224 ± 0.004 ^d^	0.222 ± 0.006 ^d^	0.238 ± 0.007 ^c^	0.258 ± 0.003 ^b^	0.268 ± 0.002 ^a^

Note: The data are expressed in the form of mean ± standard deviations (n = 5). Different letters (a–f) within the same row indicate significant differences (*p* < 0.05) between mean values.

## Data Availability

Not applicable.

## Supplementary Materials

The following supporting information can be downloaded at: https://www.mdpi.com/article/10.3390/gels8020106/s1, Figure S1: Effects of safflower seed oil on water-holding capacity and cooking loss rate of the *Nemipterus virgatus* surimi gel.; Table S1: Effect of pre-emulsified safflower seed oil on volatile compound and content of the *N. virgatus* surimi gel (μg/kg Gel).

## Abbreviations

water-holding capacity	WHC
cooking loss rate	CLR
whey protein isolate	WPI
ethylenediaminetetraacetic acid	EDTA
low-field nuclear magnetic resonance	LF-NMR
Carr–Purcell–Meiboom–Gill	CPMG
scanning electron microscopy	SEM
Fourier-transform infrared spectroscopy	FT-IR
thiobarbituric acid reaction substances	TBARS
malondialdehyde	MDA
headspace solid-phase microextraction–gas chromatography–mass spectrometry	HS-SPMEGC-MS
standard deviation	SD
