# Supplementary material for: Low-Content Pre-Emulsified Safflower Seed Oil Enhances the Quality and Flavor of the Nemipterus Virgatus Surimi Gel
Source: Gels. 2022 Feb 9;8(2):106. doi: 10.3390/gels8020106 (PMC8871502; doi:10.3390/gels8020106)
Supplement: Supplementary file 1 [file gels-08-00106-s001.zip › gels-1567930-supplementary.pdf]

Supplementary materials

Preparation of surimi gel

After thawing at 4 °C overnight, the surimi was cut into small pieces. Salts (2.5 g/100 g) were added to surimi and chopped at a speed of 2100 rpm for 2 min in a Stephan vertical vacuum cutter (Model UM 5; Stephan Machinery Co., Hameln, Germany). Subsequently, safflower seed oils were added into salted surimi pastes, and the final moisture contents were adjusted to 80% with ice water, chopping at the speed of 2100 rpm for 3 min. The final contents of safflower seed oil in the surimi were 1, 2, 3, 4, and 5 mL per 100 g surimi, respectively. During chopping, water was used as a cooling medium to keep the sample temperature below 8 °C. After eliminating the air pockets, surimi was poured into plastic casing with a diameter of 2.5 cm and sealed at both ends. Finally, samples were set at 40 °C for 30 min and then placed in a water bath at 90 °C for 20 min [1,13]. After water bath heating, samples were immediately put in ice water and then stored at 4 °C. Surimi gel without safflower seed oil was the control.

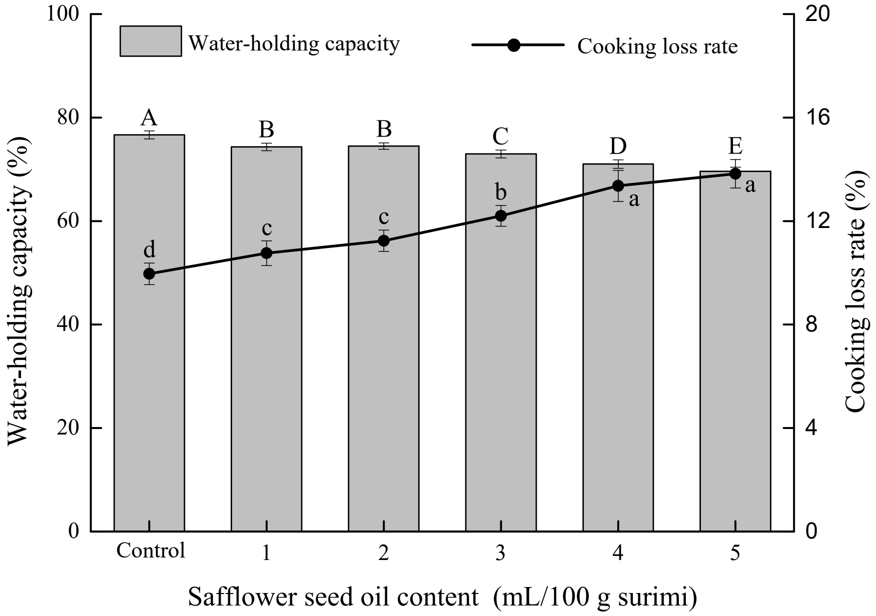

Figure S1. Effects of safflower seed oil on water-holding capacity and cooking loss rate of the *N. virgatus* surimi gel.

Table S1. Effect of pre-emulsified safflower seed oil on volatile compounds and content of the *N. virgatus* surimi gel (μg/kg Gel).

Retent Time (min)	Volatile Compounds	Identification Method	RI	Control	Safflower Seed Oil Content (mL/100 g surimi)				
					1	2	3	4	5
3.449	Hexanal	MS		5.59 ± 0.59[d]	15.37 ± 0.61[c]	15.85 ± 0.53[c]	19.35 ± 1.80[b]	20.29 ± 0.30[b]	26.34 ± 0.66[a]
5.335	1-Hexanol	MS		0.65 ± 0.06[d]	1.18 ± 0.13[c]	1.44 ± 0.15[b]	1.89 ± 0.05[a]	1.88 ± 0.06[a]	1.89 ± 0.04[a]
6.023	2-Heptanone	MS		0.60 ± 0.06[d]	1.62 ± 0.14[c]	1.67 ± 0.04[c]	1.92 ± 0.08[b]	2.34 ± 0.06[a]	2.38 ± 0.05[a]
6.472	Heptanal	MS		—	—	—	—	8.87 ± 0.37[a]	8.79 ± 0.31[a]
7.531	Thiazole, 4,5-dimethyl-	MS		—	—	—	—	0.05 ± 0.02[a]	0.05 ± 0.02[a]
8.568	Benzaldehyde	MS		0.76 ± 0.05[e]	1.34 ± 0.06[d]	1.83 ± 0.06[c]	2.56 ± 0.58[b]	2.89 ± 0.06[ab]	3.09 ± 0.05[a]
9.115	2-Hexene, 3,5,5-trimethyl-	MS, RI	972	—	4.71 ± 0.40[a]	4.61 ± 0.46[a]	4.62 ± 0.24[a]	4.64 ± 0.20[a]	4.69 ± 0.27[a]
9.396	1-Octen-3-ol	MS, RI	1001	4.71 ± 0.58[c]	6.29 ± 0.60[b]	6.52 ± 0.76[b]	7.98 ± 0.67[a]	8.50 ± 0.29[a]	8.81 ± 0.31[a]

9.549	2,5-Octanedione	MS, RI	1006	5.17 ± 0.57^{c}	6.42 ± 0.31^{b}	7.34 ± 1.13^{ab}	6.68 ± 0.42^{b}	8.24 ± 0.14^{a}	8.22 ± 0.09^{a}
10.231	Octanal	MS, RI	1029	2.57 ± 0.25^{c}	3.27 ± 0.15^{b}	3.12 ± 0.30^{b}	3.26 ± 0.19^{b}	4.15 ± 0.11^{a}	4.16 ± 0.21^{a}
11.169	1-Hexanol, 2-ethyl-	MS, RI	1061	2.34 ± 0.37^{a}	2.28 ± 0.12^{a}	2.11 ± 0.04^{a}	2.13 ± 0.19^{a}	1.46 ± 0.07^{b}	1.75 ± 0.14^{b}
11.408	4-Ethylcyclohexanol	MS, RI	1069	—	—	—	0.42 ± 0.07^{a}	0.27 ± 0.02^{b}	0.27 ± 0.02^{b}
12.168	Decane, 1,1′-oxybis-	MS		—	0.29 ± 0.03^{ab}	0.26 ± 0.01^{b}	0.25 ± 0.06^{b}	—	0.33 ± 0.03^{a}
12.214	Trichloroacetic acid, decyl ester	MS		—	—	—	0.35 ± 0.03^{a}	—	—
12.564	2-Octen-1-ol, (E)-	MS, RI	1108	0.36 ± 0.05^{c}	0.72 ± 0.02^{b}	1.35 ± 0.30^{a}	1.39 ± 0.16^{a}	1.51 ± 0.05^{a}	1.41 ± 0.05^{a}
12.635	3-Cyclohexene-1-ethanol	MS, RI	1110	1.55 ± 0.15^{a}	—	—	—	—	—
13.002	4-Hexen-1-ol, acetate	MS, RI	1123	0.46 ± 0.03^{c}	—	0.39 ± 0.10^{c}	1.54 ± 0.34^{b}	1.98 ± 0.06^{a}	1.98 ± 0.06^{a}
13.371	2-Nonanone	MS, RI	1086	1.60 ± 0.25^{a}	1.23 ± 0.12^{a}	0.72 ± 0.14^{c}	0.63 ± 0.08^{cd}	0.42 ± 0.03d^{e}	0.36 ± 0.02^{e}
13.420	1-Nonen-4-ol	MS, RI	1088	—	—	—	1.21 ± 0.04^{a}	—	—
13.738	Undecane	MS, RI	1099	0.64 ± 0.06^{d}	0.86 ± 0.03^{c}	1.10 ± 0.08^{b}	0.88 ± 0.03^{c}	1.77 ± 0.02^{a}	1.72 ± 0.01^{a}
13.835	Nonanal	MS, RI	1099	—	7.51 ± 0.38^{a}	7.02 ± 0.27^{ab}	7.76 ± 0.70^{a}	5.35 ± 0.11^{c}	6.23 ± 0.71^{b}
14.008	Phenylethyl Alcohol	MS, RI	1157	1.06 ± 0.13^{d}	1.37 ± 0.10^{cd}	2.13 ± 0.49^{b}	1.56 ± 0.10^{c}	2.28 ± 0.12^{b}	2.78 ± 0.18^{a}
14.171	6-Tridecen-4-yne, (Z)-	MS, RI	1119	1.02 ± 0.11^{ab}	1.18 ± 0.20^{a}	1.23 ± 0.33^{a}	0.84 ± 0.05^{b}	0.53 ± 0.05^{c}	—
14.684	1-Hexadecanol	MS, RI	1021	0.04 ± 0.01^{a}	—	—	—	—	—
15.706	2-Nonenal, (E)-	MS, RI	1149	0.12 ± 0.02^{d}	0.14 ± 0.01^{d}	0.14 ± 0.02^{cd}	0.22 ± 0.03^{a}	0.18 ± 0.01^{bc}	0.19 ± 0.01^{ab}
15.729	Benzaldehyde, 4-ethyl-	MS, RI	1150	0.16 ± 0.03^{a}	0.14 ± 0.01^{ab}	0.10 ± 0.03^{c}	0.13 ± 0.01^{bc}	—	—
15.885	1,2-Propanedione, 1-phenyl-	MS, RI	1154	—	—	0.10 ± 0.05^{a}	0.06 ± 0.03^{b}	—	—
16.111	13-Methyltetradecanal	MS, RI	1159	0.71 ± 0.04^{a}	0.19 ± 0.03^{b}	0.18 ± 0.04^{b}	0.15 ± 0.02^{bc}	0.11 ± 0.01^{c}	0.10 ± 0.01^{c}
16.174	1-Nonanol	MS, RI	1161	—	0.16 ± 0.02^{a}	0.08 ± 0.01^{c}	0.13 ± 0.01^{b}	—	—
16.865	6-Dodecene, (E)-	MS, RI	1184	—	—	0.12 ± 0.01^{a}	0.09 ± 0.02^{b}	0.13 ± 0.02^{a}	0.13 ± 0.02^{a}
16.976	2-Decanone	MS, RI	1190	0.08 ± 0.02^{b}	0.09 ± 0.01^{b}	0.08 ± 0.02^{b}	0.08 ± 0.01^{b}	0.07 ± 0.01^{b}	0.12 ± 0.03^{a}
17.059	4-Decenal, (E)-	MS, RI	1192	—	0.19 ± 0.02^{a}	—	—	—	—
17.141	1,3-Cyclooctadiene	MS, RI	1263	0.45 ± 0.06^{c}	0.68 ± 0.06^{b}	0.75 ± 0.17^{b}	0.75 ± 0.10^{b}	1.44 ± 0.04^{a}	1.44 ± 0.04^{a}
17.428	Dodecane	MS, RI	1199	0.37 ± 0.03^{b}	0.44 ± 0.02^{a}	0.43 ± 0.05^{ab}	0.44 ± 0.04^{a}	0.44 ± 0.03^{a}	0.47 ± 0.01^{a}
17.589	Decanal	MS, RI	1207	4.08 ± 0.28^{a}	2.30 ± 0.33^{b}	1.40 ± 0.13^{c}	1.17 ± 0.18^{cd}	0.76 ± 0.05^{e}	0.87 ± 0.09^{de}
19.302	Caprolactam	MS		0.14 ± 0.02^{d}	0.50 ± 0.05^{c}	0.47 ± 0.03^{c}	0.65 ± 0.08^{ab}	0.62 ± 0.06^{b}	0.73 ± 0.02^{a}
20.068	3-Dodecyne	MS, RI	1269	0.99 ± 0.09^{d}	1.24 ± 0.12^{cd}	1.50 ± 0.34^{bc}	1.60 ± 0.07^{b}	1.94 ± 0.09^{a}	2.10 ± 0.14^{a}
20.078	cis,cis-7,10,-Hexadecadienal	MS, RI	1197	—	—	—	—	0.58 ± 0.05^{a}	—
20.869	Indole	MS, RI	1389	0.19 ± 0.04^{d}	0.23 ± 0.01^{cd}	0.34 ± 0.07^{bc}	0.24 ± 0.02^{cd}	0.53 ± 0.04^{a}	0.33 ± 0.05^{ab}
21.077	2-Undecanone	MS, RI	1297	0.69 ± 0.08^{c}	0.79 ± 0.08^{bc}	0.86 ± 0.33^{bc}	0.67 ± 0.05^{c}	1.00 ± 0.12^{b}	1.34 ± 0.03^{a}
21.462	Tridecane	MS, RI	1308	0.14 ± 0.02^{b}	0.20 ± 0.02^{b}	0.18 ± 0.05^{b}	0.16 ± 0.02^{b}	0.23 ± 0.01^{a}	0.25 ± 0.01^{a}
21.661	Undecanal	MS, RI	1313	0.34 ± 0.04^{a}	0.14 ± 0.01^{b}	0.14 ± 0.02^{b}	0.15 ± 0.02^{b}	0.09 ± 0.01^{c}	0.10 ± 0.01^{c}
22.266	Dodecane, 4,6-dimethyl-	MS, RI	1317	0.05 ± 0.01^{c}	0.13 ± 0.02^{a}	0.09 ± 0.02^{b}	0.09 ± 0.01^{b}	0.08 ± 0.01^{b}	0.09 ± 0.01^{b}
23.711	1,11-Dodecadiyne	MS, RI	1365	—	0.21 ± 0.03^{a}	—	0.18 ± 0.03^{b}	0.16 ± 0.01^{b}	0.15 ± 0.01^{b}
24.081	2-Bromo dodecane	MS, RI	1374	—	—	—	0.02 ± 0.01^{a}	0.02 ± 0.01^{a}	0.04 ± 0.01^{a}
24.874	2-Pentadecen-4-yne, (Z)-	MS, RI	1396	0.13 ± 0.03^{a}	0.15 ± 0.01^{a}	0.15 ± 0.05^{a}	0.15 ± 0.03^{a}	0.11 ± 0.01^{a}	0.12 ± 0.01^{a}
25.170	Tetradecane	MS, RI	1400	0.27 ± 0.03^{c}	0.40 ± 0.04^{bc}	0.39 ± 0.15^{bc}	0.42 ± 0.05^{b}	0.68 ± 0.04^{a}	0.70 ± 0.05^{a}
25.405	Dodecanal	MS, RI	1409	0.23 ± 0.03^{a}	0.15 ± 0.01^{b}	0.15 ± 0.01^{b}	0.12 ± 0.02^{c}	0.11 ± 0.01^{c}	0.10 ± 0.02^{c}
25.842	1H-Indene, octahydro-2,2,4,4,7,7-hexamethyl-, trans-	MS, RI	1422	0.11 ± 0.02^{a}	0.12 ± 0.01^{a}	0.09 ± 0.03^{a}	0.09 ± 0.01^{a}	0.08 ± 0.01^{a}	0.08 ± 0.01^{a}

26.701	5,9-Undecadien-2-one, 6,10-dimethyl-, (E)-	MS, RI	1439	0.55 ± 0.03[a]	0.19 ± 0.02[b]	0.17 ± 0.06[b]	0.22 ± 0.07[b]	0.17 ± 0.01[b]	0.15 ± 0.03[b]
27.130	Decahydro-1,1,4a,5,6-pentamethylnaphthalene	MS, RI	1457	—	0.10 ± 0.02[a]	0.09 ± 0.02[a]	0.09 ± 0.01[a]	0.06 ± 0.02[a]	0.07 ± 0.02[a]
27.254	2,6,10-Trimethyltridecane	MS, RI	1431	0.09 ± 0.01[b]	0.12 ± 0.01[a]	0.11 ± 0.03[ab]	0.13 ± 0.01[a]	0.08 ± 0.01[b]	0.08 ± 0.01[b]
28.054	7-Pentadecyne	MS, RI	1482	—	0.11 ± 0.02[b]	0.17 ± 0.07[a]	0.21 ± 0.03[a]	0.22 ± 0.01[a]	0.19 ± 0.02[a]
28.419	1-Eicosanol	MS		—	0.08 ± 0.01[a]	0.09 ± 0.04[a]	—	—	0.06 ± 0.01[a]
28.429	1-Hexadecanol, 2-methyl-	MS, RI	1468	0.05 ± 0.01[a]	—	—	0.06 ± 0.01[a]	0.05 ± 0.01[a]	0.04 ± 0.01[a]
28.729	Pentadecane	MS, RI	1500	1.51 ± 0.31[c]	1.92 ± 0.27[b]	1.70 ± 0.71[b]	2.01 ± 0.04[a]	2.37 ± 0.13[a]	2.33 ± 0.07[a]
29.023	Tributyl phosphate	MS		0.41 ± 0.04[a]	0.33 ± 0.02[a]	0.34 ± 0.15[a]	0.45 ± 0.09[a]	0.38 ± 0.01[a]	0.37 ± 0.03[a]
30.683	1,8,11-Heptadecatriene, (Z,Z)-	MS, RI	1542	—	—	0.06 ± 0.03[b]	0.09 ± 0.01[a]	—	—
30.703	10(E),12(Z)-Conjugated linoleic acid	MS		—	—	—	—	0.08 ± 0.01[a]	0.06 ± 0.01[a]
30.770	1,6,10-Dodecatrien-3-ol, 3,7,11-trimethyl-, (E)-	MS, RI	1555	—	0.10 ± 0.01[a]	—	—	0.09 ± 0.01[a]	0.09 ± 0.01[a]
31.686	2,2,4-Trimethyl-1,3-pentanediol diisobutyrate	MS, RI	1576	0.32 ± 0.04[cd]	0.70 ± 0.10[a]	0.51 ± 0.08[b]	0.38 ± 0.04[c]	0.38 ± 0.02[c]	0.26 ± 0.02[d]
32.567	Tetradecanal	MS, RI	1609	0.12 ± 0.02[a]	0.13 ± 0.03[a]	0.12 ± 0.04[a]	0.13 ± 0.01[a]	0.06 ± 0.01[b]	0.07 ± 0.01[b]
32.870	Phenol, 2,6-bis(1,1-dimethylethyl)-4-(1-methylpropyl)-	MS		0.05 ± 0.01[a]	0.08 ± 0.03[a]	0.07 ± 0.02[a]	0.07 ± 0.01[a]	0.05 ± 0.01[a]	0.06 ± 0.01[a]
34.635	1,2-Oxathiane, 6-dodecyl-, 2,2-dioxide	MS, RI	1672	—	0.07 ± 0.01[c]	0.07 ± 0.02[c]	—	0.43 ± 0.04[a]	0.30 ± 0.03[b]
35.420	Hexadecane	MS, RI	1609	0.18 ± 0.02[b]	0.25 ± 0.03[a]	0.24 ± 0.02[a]	0.26 ± 0.02[a]	0.20 ± 0.01[a]	0.22 ± 0.01[a]
35.548	Pentadecane, 2,6,10,14-tetramethyl-	MS, RI	1755	0.70 ± 0.07[b]	—	—	0.87 ± 0.03[a]	—	—
35.783	10-Octadecenal	MS, RI	1844	—	—	0.09 ± 0.01[a]	—	0.07 ± 0.01[a]	—
35.790	Tridecanal	MS, RI	1670	0.12 ± 0.01[a]	—	—	0.12 ± 0.01[a]	0.09 ± 0.01[b]	0.09 ± 0.01[b]
38.370	Heptadecane	MS, RI	1773	—	0.03 ± 0.01[a]	0.03 ± 0.02[a]	0.03 ± 0.01[a]	—	0.03 ± 0.02[a]
38.817	Hexadecanal	MS, RI	1809	0.16 ± 0.02[b]	0.20 ± 0.02[a]	0.12 ± 0.01[c]	0.17 ± 0.02[b]	—	0.10 ± 0.00[c]
39.234	4,8,12-Tetradecatrienal, 5,9,13-trimethyl-	MS, RI	1823	—	0.06 ± 0.01[a]	—	—	0.06 ± 0.02[a]	—
40.065	1,2-Benzenedicarboxylic acid, bis(2-methylpropyl) ester	MS, RI	1850	—	0.28 ± 0.03[c]	0.27 ± 0.04[c]	0.52 ± 0.14[b]	0.68 ± 0.04[a]	0.71 ± 0.06[a]
41.756	1-Heptadecanamine, N,N-dimethyl-	MS		—	—	—	—	—	0.05 ± 0.01[a]
41.769	Dimethyl palmitamine	MS		0.04 ± 0.01[a]	0.06 ± 0.02[a]	0.06 ± 0.01[a]	0.05 ± 0.01[a]	0.04 ± 0.01[a]	0.04 ± 0.01[a]
43.002	Dibutyl phthalate	MS, RI	1945	0.25 ± 0.03[c]	0.29 ± 0.02[c]	0.45 ± 0.17[b]	0.46 ± 0.08[b]	0.63 ± 0.04[a]	0.65 ± 0.05[a]

Note: The data are expressed in the form of mean ± standard deviations (n = 3). Different letters (a–e) within the same row indicate significant differences ($P < 0.05$) between mean values, "—" means not detected.
